# The influence of self-stretch based on post-isometric relaxation, static stretch combined with stabilization exercises and stabilization exercises only on hamstring, one-joint and two-joints hip flexors flexibility and finger-to-floor test results

**DOI:** 10.1186/1748-7161-8-S1-P1

**Published:** 2013-06-03

**Authors:** D Czaprowski, Leszczewska, A Kolwicz, P Pawłowska, T Kotwicki, L Stoliński, A Kędra

**Affiliations:** 1 Department of Physiotherapy, Józef Rusiecki University College in Olsztyn, Olsztyn, Poland; 2 Department of Pediatric Orthopedics and Traumatology, University of Medical Sciences in Poznań, Poland; 3 Rehasport Clinic, Poznań, Poland; 4 Faculty of Physical Education and Sport in Biala Podlaska, Józef Piłsudski University of Physical Education in Warsaw, Poland

## Background

Stretching techniques are commonly used in clinical practice to increase flexibility of muscles in children with idiopathic scoliosis, as well as with faults of body posture. The proper flexibility of muscles is important in prevention of postural imbalance, maintaining of full range of motion, or in decrease of risk of back pain.

## Aim

Prospective randomized single-blinded evaluation of changes of hamstring, one-joint and two-joint hip flexors flexibility in children under the 6-week therapeutic programme consisting of one physiotherapy session per week and daily home exercises.

## Methods

94 children (46 boys and 48 girls) aged 10-13 years (11.5 ±0.5), were randomly divided into 3 groups. Each group underwent a 6-week therapeutic programme aiming to correct muscles flexibility. The first group-GI (31 children) realized the programme of post isometric muscle relaxation (PIR), the second one-GII (31 children) performed static stretching with stabilization exercises, the third group-GIII (32 children) realized only stabilization exercises. The shortening of hamstring, one-joint and two-joint hip flexors was assessed clinically according to Kendall. The straight leg raise (SLR test) angle and the popliteal angle were measured for hamstrings, the angle in sagittal plane in hip for one-joint and the knee flexion was measured for two-joint hip flexors. Fingertips-to-floor distance (FTF test) in trunk flexion was also noted. The examination was conducted twice – before therapy and a week after its completion, by the blinded observers.

## Results

No difference in the amount of correction of muscle flexibility among the three groups was found for hamstrings (p=0.14), one-joint (p=0.38) and two-joint hip flexors (p=0.28) as well as for FTF test (p=0.15). Significant correction of all muscle flexibility and FTF test (p<0.05) was observed in GI and GII groups. In GIII significant improvement (p<0.05) in SLR test and flexibility of one-joint hip flexors was obtained.

## Conclusions

Postisometric muscle relaxation, static stretching with stabilization exercises, as well as only stabilization training had similar influence on the improvement of muscle flexibility. Body suppleness, as assessed by fingertips-to-floor distance, was improved only after used techniques included stretching exercises (PIR, static stretching), but there were no differences between all three groups regarding changes in FTF test. Regardless of the stretching methods, a 6–week physiotherapeutic procedure resulted in increased flexibility of the muscles of the pelvic girdle.
